# The Key Drivers of Brain Injury by Systemic Inflammatory Responses after Sepsis: Microglia and Neuroinflammation

**DOI:** 10.1007/s12035-022-03148-z

**Published:** 2022-11-29

**Authors:** Yuewen Xin, Mi Tian, Shuixiang Deng, Jiaying Li, Miaoxian Yang, Jianpeng Gao, Xu Pei, Yao Wang, Jiaying Tan, Feng Zhao, Yanqin Gao, Ye Gong

**Affiliations:** Department of Critical Care Medicine of Huashan Hospital, State Key Laboratory of Medical Neurobiology, MOE Frontiers Center for Brain Science and Institutes of Brain Science, Fudan University, Shanghai, China

**Keywords:** Sepsis-associated encephalopathy, Cognitive impairment, Synaptic dysfunction, Mitochondrial dysfunction, Blood–brain barrier

## Abstract

Sepsis is a leading cause of intensive care unit admission and death worldwide. Most surviving patients show acute or chronic mental disorders, which are known as sepsis-associated encephalopathy (SAE). Although accumulating studies in the past two decades focused on the pathogenesis of SAE, a systematic review of retrospective studies which exclusively focuses on the inflammatory mechanisms of SAE has been lacking yet. This review summarizes the recent advance in the field of neuroinflammation and sheds light on the activation of microglia in SAE. Activation of microglia predominates neuroinflammation. As the gene expression profile changes, microglia show heterogeneous characterizations throughout all stages of SAE. Here, we summarize the systemic inflammation following sepsis and also the relationship of microglial diversity and neuroinflammation. Moreover, a collection of neuroinflammation-related dysfunction has also been reviewed to illustrate the possible mechanisms for SAE. In addition, promising pharmacological or non-pharmacological therapeutic strategies, especially those which target neuroinflammation or microglia, are also concluded in the final part of this review. Collectively, clarification of the vital relationship between neuroinflammation and SAE-related mental disorders would significantly improve our understanding of the pathophysiological mechanisms in SAE and therefore provide potential targets for therapies of SAE aimed at inhibiting neuroinflammation.

## Introduction

Sepsis, which is initially caused by infection, is characterized by an excessive systemic inflammatory response. Following this uncontrollable process, innate immune homeostasis can be destroyed, causing terminal organ dysfunction, which is referred to as systemic inflammatory response syndrome (SIRS) [1]. Brain could be first affected during sepsis, manifesting with acute or chronic brain injury, which is currently known as sepsis-associated encephalopathy (SAE). A total of 32.3% of SAE patients present with mental disorders such as delirium or depression [2]. The etiology of SAE is multifactorial, ischemic/hemorrhagic lesions, blood–brain barrier (BBB) destruction, cerebrovascular dysfunction, and metabolic changes are the main pathomechanisms involved in the development of SAE [3]. All of these mechanisms are collectively mediated by a variety of immune factors, which orchestrate genetic program that controls diverse cellular and molecular pathways, including immune response pathways [4]. In terms of sepsis, inflammatory responses in the brain can be divided into two categories: primary inflammatory responses induced by resident immune cells and secondary inflammatory responses induced by peripheral immune cells. Resident immune cells, which are represented by a large group of marcophage/microglia, constitute the brain's main self-defense power. Microglia are intrinsic myeloid parenchymal cells in the central nervous system (CNS) and are the most abundant cells in the mononuclear phagocyte system [5]. The dominant function of microglia in resting state is to monitor the normal functioning of the brain and maintain CNS homeostasis. Nevertheless, under the background of infection or brain injury, they display dramatic morphological transition in response to Damage-associated molecular patterns (DAMPs) and initiate primary inflammatory response. Excessive local inflammatory activation of innate immune cells in the brain can detrimentally mediate brain damage. Subsequently, peripheral immune cells are recruited prior to larger-scale activation of cerebral endothelial cells (ECs) and microglia to initiate secondary inflammatory responses. In the septic context of violent secretion of inflammatory factors and chemokines, peripheral immune cells, such as neutrophils, macrophages, can be recruited to prompt the breakdown of BBB and thereby aggravate cerebral damage. Studies have shown that neutrophils are the main leukocytes recruited to lesions during infection or tissue damage. Increased expression levels of inflammatory mediators in these immune cells, such as TNF-α, IL-1β, and IL-6, might amplify the response of microglia and glial cells activation, and thus exacerbate neuroinflammation. A considerable amount of evidence from clinical or experimental research has indicated that systemic immune signaling originating inside or outside the brain constitute the main power to aggravate brain injury in sepsis [6, 7]. Therefore, neuroinflammation can be considered as a central player in SAE pathogenesis.

In this review, we primarily focus on the influences of sepsis-induced systemic inflammatory responses and cytokine storms on the brain, with the aim of explaining how cytokines enter the brain and trigger neuroinflammation. Then, we discuss the role of microglia in SAE, to depict the activation of these key agents in neuroinflammation and how these brain-resident immune cells present with heterogeneous transition in the inflammatory environment, aiming to gain a more comprehensive understanding of neuroinflammation development in SAE. Specifically, we provide an overview of recent reports that describe how context-distinct diversity of microglia occurs over time and its involvement in pathology at different stages of disease. These studies facilitate our understanding of the correlation between multiple parallel pathological factors of septic brain injury in both humans and mice, which might provide theoretical guidance for future clinical treatment.

## Cytokine Storm and the Innate Immune Response in the SAE

The systemic inflammatory response initiated by sepsis leads to damage to multiple target organs, which is an important prerequisite for neuroinflammation. Following a devastating infection, the causative pathogens are recognized by host immune cells, which trigger the initial inflammatory response [8, 9]. Both proinflammatory and anti-inflammatory mediators released by this pattern recognition are thought to be critical for an uncontrollable secretion of cytokines [10]. Two types of signaling patterns are recognized by pattern recognition receptors (PRRs) in innate immune cells, which trigger the inflammatory response [11]. One type comprises pathogen-associated molecular patterns (PAMPs), which consist of structural components and/or metabolites produced by invading microorganisms. PAMPs mainly include LPS, bacterial flagellin, lipoprotein acids, peptidoglycan, and virus double-stranded RNA or unmethylated CpG motifs [12]. The other type comprises DAMPs, which are mainly endogenous host components released by injured cells [12]. High mobility group box 1 (HMGB1), histones, sequestosome 1 (SQSTM1), heat shock protein family A (Hsp70) member 5 (HSPA5), mitochondrial transcription factor A (mtTFA), ATP and DNA are commonly regarded as DAMPs [12]. Innate immune cells can be activated once PRRs have been bound. The downstream intracellular signaling cascades can lead to the activation of transcription factors, such as NF-κB, activator protein-1 and interferon regulatory factor [13]. Mostly, the innate immune system can produce a balanced immune response to eliminate pathogens, following by return to homeostasis [13]. However, in sepsis, excessive immune response can be harmful. The current study support that the early proinflammatory response of sepsis can be overwhelming, characterized by excessive release of proinflammatory cytokines, leading to multi-target organ damage [13]. Whereas in a later phase an unbalanced anti-inflammatory response can occur, which may be the basis for further infection or immune dysfunction of patients.

### Pro-inflammatory Cytokine Response after Sepsis

The cytokine signaling cascade caused by the early excessive release of proinflammatory cytokines after sepsis can be also called cytokine storm. Numerous cytokines, such as TNF-α, IL-1, IL-2, IL-6, and IL-18, are linked with the extensive activation of innate immune responses induced by the activation of NF-κB and neutrophils recruitment [14]. Although proper activation of the innate immune system can prompt pathogen elimination, excessive activation of the innate immune system causes detrimental effects that lead to tissue damage and/or infection acceleration. Reports that investigate several clinical samples have described cytokine storm in the brain or other peripheral organs and provided critical insights into the roles of different cytokines in the progression of sepsis [15, 16]. For example, TNF-α has been proven to play a key role in orchestrating activation of cytokine cascades in several inflammatory diseases and has been implicated in the development of cognitive impairment in the brains of Alzheimer's disease (AD) patients [17–19]; IL-1 contributes to the impairment of hippocampal-dependent memory because of its impact on long-term potentiation (LTP), which underlies the mechanism in the hippocampal pathway [20, 21]; IL-6 can increase vascular permeability, and facilitate lymphocyte activation and antibody synthesis, eventually leading to CNS disorders [22, 23]; IL-18 is a well-known mediator of TNF-α activity and functions synergistically with IL-1β to facilitate inflammasome activity in neuroinflammation-induced neurodegeneration [24, 25]. Taken together, these studies suggested that inflammatory cytokine storms triggered by PRRs recognition which are activated by DAMPs and/or PAMPs play a crucial role in the multiple organ dysfunction observed after sepsis infection.

### Anti-inflammatory Cytokine Response after Sepsis

Anti-inflammatory cytokine response in a later stage of sepsis requires the participation of immune cells with inhibitory functions, including myeloid derived suppressor cells (MDSCs), regulatory T cells, innate-type lymphocytes, dendritic cells, and macrophages [16]. With an altered innate and adaptive immune functions after sepsis, a large number of anti-inflammatory cytokines were generated to constitute the ‘immunosuppression’. It is characterized by decreased secretion of proinflammatory cytokines and increased secretion of anti-inflammatory cytokines. For example, IL-10, mainly produced by MDSCs, regulatory T cells, inhibits the proinflammatory activation of microglia and macrophages without stimulating inflammatory CD8^+^T cells, and reduces cell death under the regulation of inflammatory response [26, 27]; TGFβ, produced by MDSCs and innate-type lymphocytes, results in inhibition of hyperpermeability, expression of cell adhesion molecules, and adhesion and migration of leukocytes [28]. The current research suggests that the host’s immunosuppressive response could be a possible reason for uncontrolled neuroinflammation [29]. Uncontrolled neuroinflammation and intervention of brain perfusion lead to irreversible brain damage, thus forming a vicious cycle of SAE immunosuppression [29].

### The Pathways of Cytokines Entering the Brain after Sepsis

**Fig. 1:**
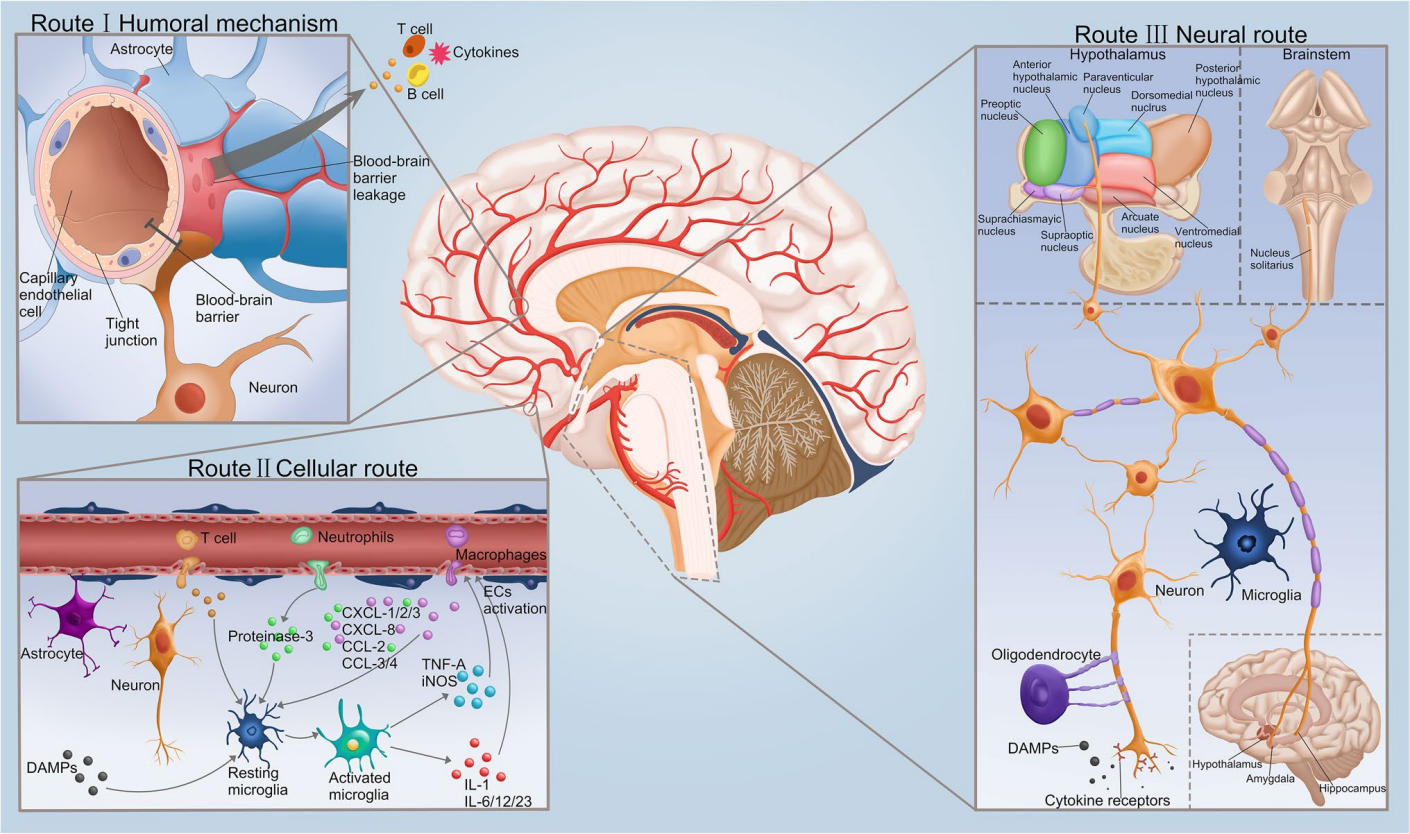
Routes of cytokines enter the brain. Route I: The systemic inflammatory response induced by sepsis causes extensive cerebral vascular endothelial cell inflammatory response, which may be responsible for the destruction of the BBB. This route is known as the humoral route or humoral mechanism. Peripheral immune cells and cytokines enter the brain parenchyma through the leaked BBB. Route II: Peripheral infiltrating immune cells exacerbate BBB damage. Microglia are activated to release cytokines and adhesion molecules, which cooperate with infiltrating cells to further aggravate BBB damage. This is known as the cellular route. Route III: The leakage of BBB results in the entry of cytokines into the brain parenchyma, where they are subsequently recognized by cytokine receptors expressed on neurons and glial cells in the limbic system, brain stem, and hypothalamic-pituitary system. Cytokine receptors on afferent nerve fibers and transmit cytokine signals to the brain. This process is facilitated and assisted by CNS resident inflammatory cells. **Abbreviations:** BBB, blood–brain barrier; CXCL-1/2/3, chemokine (C-X-C motif) ligand 1/2/3 protein; CCL-2, chemokine (C–C Motif) ligand 2; DAMP, damage-associated molecular pattern; EC, endothelial cell; G-CSF, granulocyte colony-stimulating factor; GM-CSF, granulocyte–macrophage colony-stimulating factor; IL, interleukin; TNF-α: tumor necrosis factor α

The findings showing that postsepsis cytokine storms are associated with acute brain damage and/or long-term cognitive impairment, possibly in a cause-and-effect manner with translational implications, have inspired an increasing number of studies directed to discovering how peripheral inflammatory responses affect the brain. Three well-described routes allow inflammatory mediator trafficking into the brain (Fig. 1). In Route I, a humoral mechanism involves inflammatory cytokines entering the brain through physiologically deficient or leaky BBB areas, mainly in periventricular organs; circulating cytokines are actively transported into the brain parenchyma via specific carriers [30]. These cytokines then diffuse into deep regions of the brain and are recognized by cytokine receptors expressed on neurons and glial cells in various parts of the limbic, noradrenergic, vasopressinergic and/or hypothalamic-pituitary systems [31]. Moreover, cytokines have been shown to affect brain function without directly crossing the BBB; for example, they can stimulate afferent nerves, exert effects on periventricular organs, disrupt BBB function, and induce BBB secretions [32]. Route II refers to a cellular route. Activation of major inflammatory cells in the CNS releases chemokines and adhesion molecules that attract peripheral immune cells to the meninges and brain parenchyma [30]. In addition, inflammatory mediators released by activated microglia and astrocytes can cause the activation of cerebral ECs, which contributes to the destruction and leakage of the BBB, and accelerate the entry of peripheral immune cells and cytokines into the brain parenchyma [31]. Severe damage in brain areas follows invasion of cytokines, particularly within the limbic system, hypothalamus–pituitary axis, and brainstem. Route III is a neural route that involves autonomic nerve fibers; neuronal terminals sense infectious stimuli, activating cytokine receptors on afferent nerve fibers, and transmitting cytokine signals to the brain [30]. This circuit connects several brainstem nuclei and establishes neuronal projections into the supraoptic nucleus, paraventricular nucleus of the hypothalamus, amygdala and hippocampus [31]. The vagus nerve rapidly transmits immune information to the CNS, directly activating specific targets without hindrance by the BBB [32].

The CNS specifically modulates systemic inflammatory responses to endotoxin through humoral mechanisms. Lymphocytes can directly receive neural input from autonomic nerve fibers. Studies have shown that injection of LPS into the periphery can cause dendritic cells near the vagus nerve to express IL-1 and nodose ganglia neurons to express IL-1 receptors, which activate nodose ganglia neurons and transmit signals to the CNS [32]. In addition, researchers have found sympathetic postganglionic neurons, which were hypothesized to indicate the presence of nicotine acetylcholine α7 receptor-mediated anti-inflammatory effects [33]. This hypothesis supported a peripheral anti-inflammatory role for sympathetic but not for parasympathetic efferent fibers. Interestingly, stimulation of the vagus nerve has been shown to prevent cytokine-induced and endotoxin-induced systemic inflammation, because vagus nerve terminals regulate major components of the inflammasome [34].

Recently, it has been recognized that brain damage in the absence of microbial infection can also initiate innate immune activation by releasing inflammatory mediators [35]. Following the release of these cytokines, maladaptive immune reactions can aggravate inflammatory damage and ultimately result in cognitive dysfunction. However, novel treatments to block the activation of these mediators have not been well developed. A previous study showed that anaplastic lymphoma kinase (ALK), a DAMP, crucially participates in the regulation of the innate immune response after sepsis [36]. Additional research is required to better understand the rules that govern pathological vs. nonpathological innate immune reactions after DAMP signaling in the brain in sepsis.

## Neuroinflammation and the Activation of Microglia after Sepsis

The pathophysiological mechanisms of SAE characterized by brain dysfunction are thought to be associated with continuous microglial activation [37]. This section mainly focuses on the association between neuroinflammation and microglia, and depicts how microglia, which act as the main sponsor for neuroinflammation in the brain, respond to inflammatory responses and make phenotypic changes after sepsis.

### Neuroinflammation in the SAE Brain

The neuroinflammation in SAE is unambiguous. Postmortem analysis of patients who die of sepsis suggests that the expression of acute neuroinflammation markers were significantly elevated, leading to the proposal that neuroinflammation might contribute to the progression of SAE [38]. Experimental findings and neuropathological observations suggest that activation of microglia is pivotal for mediation of the behavioral effects of systemic infection [39]. Numerous signals received from the CNS environment as well as from the periphery induce microglia responses towards phenotypes to initiate a complicated neuroinflammatory response, that ultimately harm neuronal health [39]. The currently recognized process comprises signal induction by acute systemic inflammation in the CNS parenchyma that leads to the activation of microglia and glial cells. Besides, blood-derived lymphocytes and monocyte-derived macrophages simultaneously infiltrated the brain parenchyma, accompanied by frank impairment of BBB function and strong secretion of pro-inflammatory mediators [40]. The release of pro-inflammatory mediators, which include IL-1β, IL-6, IL-18, and TNF, chemokines (e.g., CCL1,CCL5 and CXCL1), small-molecular messengers (i.e., prostaglandins, NO), and reactive oxygen species, causes synaptic dysfunction and neuronal death, and inhibits neuronal regeneration [41]. SAE animal studies identified that astrocytes, and microglia play a critical role in neuroinflammation with the release of malignant inflammatory mediators in the brain parenchyma [42]. Moreover, the innate immune cells participate in this process also including capillary endothelial cells and peripheral infiltrating immune cells, especially when BBB sustains biochemical or mechanical damage [43]. Meanwhile, anti-inflammatory cytokines (e.g., IL-4, IL-10, and IL-11) are also produced during the neuroinflammation, preventing excessive pro-inflammatory response and restoring balance in the brain [44]. However, due to the excessive systemic immune response early on sepsis patients, the process of neuroinflammation can be affected by the level of global inflammation, resulting in harmful consequences for the brain.

### The Activation of Microglia: Sepsis-Associated Microglia Heterogeneity

The specific pro- or anti-inflammatory responses of microglia in ex vivo and in vivo models have been extensively studied [45]. Due to its plasticity, the homogeneity of homeostatic microglia is easily destroyed [46]. By sensing disruptions in CNS homeostasis, microglia rapidly alter their gene expression programs and functional profiles. Genome-wide transcriptional studies reveal distinct signatures in microglia against diseases [47, 48]. The alteration in gene expression is determined by disease progression, which accounts for the heterogeneous stage-dependent microglia population. They have unique molecular expression patterns, which are now known as disease-associated microglia (DAM) [49, 50]. Whereas in models of sepsis, the distinct transcriptional heterogeneity of microglia is also confirmed by single-cell transcriptomic studies. Through mimicking the systemic inflammatory and septic environment using LPS or treating primary microglia with TGF-β in vitro generates a heterogeneous population of microglia defined by specific gene signatures [51, 52]. Sousa et al. found that the expression of microglia homeostatic genes (Olfml3, Fcrl3, Tmem119, lr34, P2ry12 and Mef2c) and phagocytic genes (e.g., Tyrobp and Trem2) and anti-inflammatory genes (e.g., Mrc1 and Arg1) were significantly decreased after LPS challenge, while the classical pro-inflammatory genes were markedly increased (e.g., Il1b, Tnf, and Ccl2) [53]. By using the same approach, Shemer et al. came to similar conclusions, while gene upregulation in neurodegeneration was also observed in their study [54]. These studies demonstrated that in SAE, activated microglia are assumed to be a heterogeneous cell population. This heterogeneity is dominated by classical M1-like microglia, possibly accompanied by M2-like microglia and neurodegenerative microglia. In fact, since the distinct molecular expression patterns of microglia was associated with the different stages of disease, traditional M1 and M2-like terminology is inappropriate to describe the extensive heterogeneity of microglia. However, because of the limited research on SAE and the gene expression studies specific on SAE microglia, for the convenience of understanding, we still use traditional concept to describe microglia in this review.

#### Proinflammatory Microglia (M1-like)

During the first hour after injury or inflammation induction, microglia undergo the classic proinflammatory transition. The microglial soma size increases accompanied by enhanced immune function and weakened phagocytic function [55]. When exposed to the proinflammatory cytokines IFN-γ and TNF-α and cellular debris, the functional phenotype of the microglia is altered; that is, the cells polarize to acquire a proinflammatory (M1-like) phenotype. M1 microglia express an array of immune receptors, including toll-like receptors (TLRs), nucleotide-binding oligomerization domain (NOD)-like receptors, and scavenger receptors (SRs), that recognize noxious stimulation [56]. The immune function of proinflammatory microglia (M1-like) is induced by the release of cytokines (IL-1α, IL-1β, IL-6, IL-12, IL-23, and/or TNF-α) and the high-level expression of redox molecules (NADPH oxidase, phagocytosis oxidase and inducible nitric oxide synthase) [57]. By examining a wide range of transcriptional profiles, including LPS- or IFN-γ-induced proinflammatory markers, CCR7 and CD80 were found to be the most highly expressed markers, followed by IL-1β, IL-6, TNF-α, NOS2, COX-2, the chemokines CCL2 and CCL20, and the receptor CCR2 [55].

Proinflammatory microglia (M1-like) are the main driver of abnormal brain function in disease progression. By producing inflammatory mediators, such as cytokines, chemokines, inducible nitric oxide synthase (iNOS), IL-1β and free radicals such as ROS, microglia disrupt neuronal functions and trigger neuronal damage during sepsis [58–60]. A study of acute septic brain injury in mice has suggested that long-term cognitive decline after sepsis was associated with decreased cholinergic function and neuroinflammation, with the mice presenting with general neuron depletion and neurodegeneration in the hippocampus [58]. Evidence based on gene expression profiling and bioinformatic analysis has supported the suggestion that during neural injury, proinflammatory factors activate microglia, mediating a vicious neuroinflammatory cascade that leads to neuronal death [42]. A clinical neuropathological study on humans indicated that septic patients suffered from prominent inflammatory activation in the hippocampus, which was associated with proinflammatory microglia (M1-like) activation characterized by high NADPH oxidase and iNOS expression levels [61–63]. Data from septic mouse research have suggested that proinflammatory activation of microglia in sepsis mediates oxidative and nitrosative stress by triggering the release of redox molecules to induce cerebral cortex section damage [64]. The use of IL-4 or gadolinium chloride to inhibit the effect of proinflammatory microglia (M1-like) can lead to a decrease in the number of microglia, inhibiting the production of cytokines and preventing oxidative stress during sepsis [65]. Taken together, these findings suggested that the proinflammatory microglia subtype predominates in the brains of patients of sepsis and that the development of acute brain dysfunction after sepsis is associated with the proinflammatory response of these microglia.

#### Restorative Microglia (M2-like)

Depending on the macrophage/microglia activation state, the restorative phenotype (M2-like) can be identified during the process of inflammation. M2-like microglia mainly exert an effect on allergic responses, parasite clearance, inflammation suppression, tissue remodeling, angiogenesis, immune regulation, and tumor promotion. Moreover, restorative microglia (M2-like) have the ability to downregulate proinflammatory cytokine expression and restore immune homeostasis [66]. M2-like microglia are characterized by specific markers, such as CD163, CD206, Arg1, IL-10, PPARγ, and STAT6. Activation of signaling pathway involving TLRs, Fcy receptors, and interleukin receptors, promote microglial phenotype transition [57].

The activation of restorative microglia (M2-like) is induced by products of long-term resolution and repair function and related cytokines (IL-4, IL-10, IL-13, TGF-β, glucocorticoids, CCL2 and CXCL4) [67]. For example, restorative microglia (M2-like) downregulate inflammatory cell activation and the expression of protective extracellular matrix proteins such as Ym1/2, ornithine, as well as induce polyamine action in wound repair, and stimulate IL-10 production to increase the expression of receptors associated with phagocytosis. After being stimulated by IL-10, restorative microglia (M2-like) show an increased ability of apoptotic cell engulfment [45]. However, mouse macrophages overexpressing IL-10 do not show enhanced phagocytosis. In contrast, they exhibit a phenotype with restorative transcriptional profiles and downregulate inflammatory marker expression [68]. IL-10 has been shown to exert a protective effect on cognitive impairment [69]. When IL-10 receptors on microglia are abrogated, the homeostatic state of the microglia is disrupted and the cells are activated, permitting neuronal damage in mice [54]. Cells expressing IL-10 reported to be Ly4D^+^ natural kill (NK) cells and neutrophils prohibited excessive activation of microglia in response to peripheral LPS challenge [54]. Moreover, IL-4 regulated the restorative phenotype, as indicated by Ym1 protein and mRNA expression levels. Research has demonstrated that a reduction in the number of restorative microglia (M2-like) after inflammatory injury was observed in IL-4-deficient mice [70]. Stimulation of IL-4 and IL-13 in vitro after sepsis increases microglial mitochondrial content with its function enhanced, drives restorative microglia (M2-like) transition and inhibits the release of inflammatory cytokines to confer neuronal protective effects [71].

Recently, unresolved neuroinflammation involving a widespread microglial population has been shown to contribute to restorative effects [23]. Eliminating microglia did not mitigate cognitive impairment or neurogenesis after neuroinflammation was triggered following TBI [23]. However, early repopulation of microglia after injury attenuated learning deficits in mice by stimulating functional neurogenesis, which led to changes in morphology; thus, the morphology of microglia before and after brain injury differed.

The role of the transition and repopulation of restorative microglia (M2-like) in the brain after sepsis remains unclear. Due to the acute phase of SAE, pro-inflammatory microglia dominate the microglia population, M2-like microglia might be very rare. Therefore, to explore when exactly M2-like microglia occur on the process of neuroinflammation and participate in ameliorating cerebral damage, more research is needed to confirm.

#### Microglia Diversity during Sepsis

**Fig. 2:**
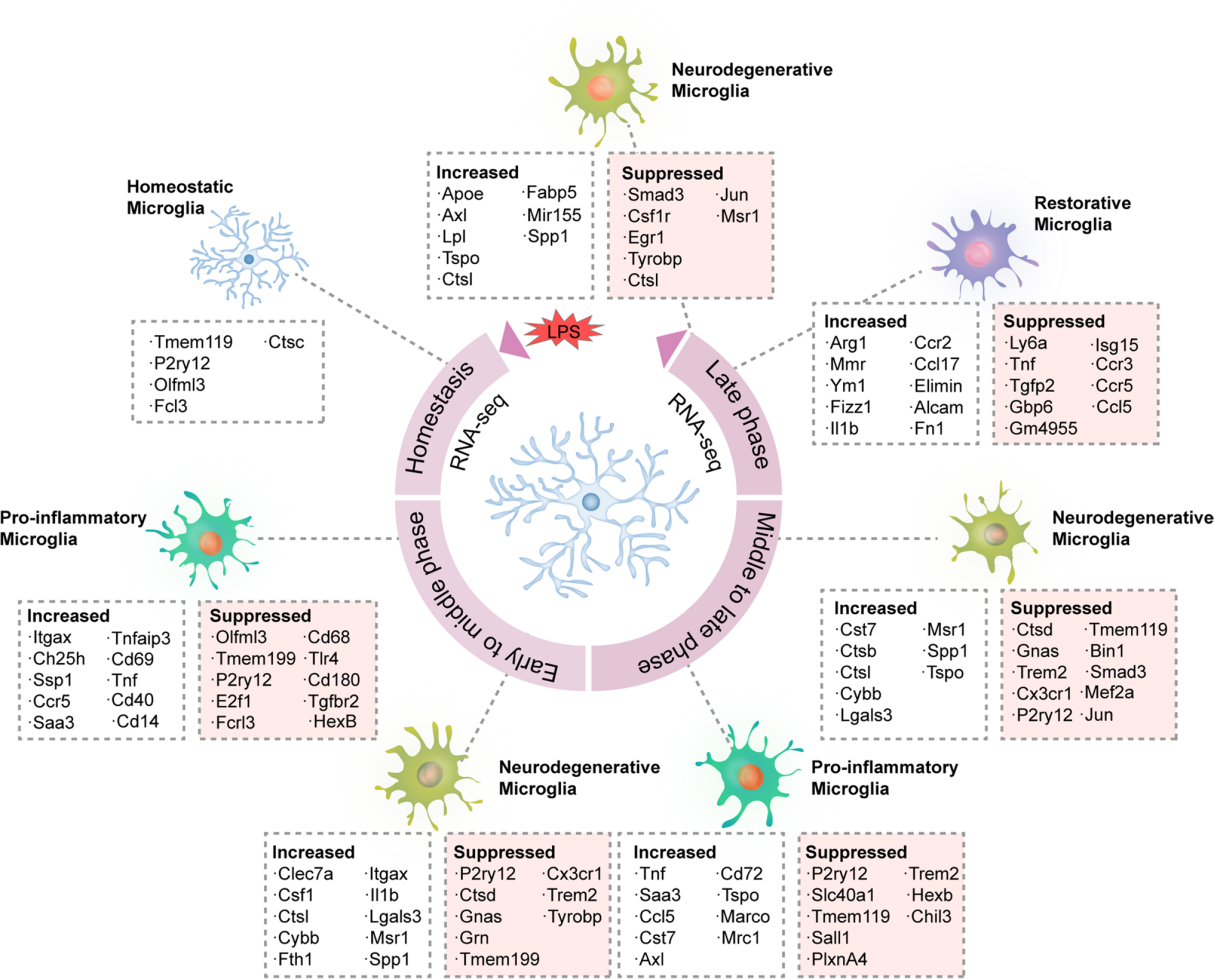
Phenotypic diversity of context-dependent microglia in the brain under conditions of inflammation mimicking sepsis after LPS challenge. Depending on the stage of disease progression, microglia acquire distinct heterogeneous phenotypes. They have unique molecular expression patterns and are defined as DAM. Through mimicking systemic inflammatory and septic environment using LPS to stimulate primary microglia in vitro, heterogeneous microglia defined by specific genetic signatures is identified by RNA-sequencing analysis at different times after LPS challenge. Microglial gene expression was analyzed by RNA sequencing (single-cell RNA-sequencing, global RNA-sequencing). Key transcriptional profiles of microglia at each phase are shown [53, 54]. Abbreviations: DAM, disease-associated microglia; LPS, lipopolysaccharides; RNA-seq, RNA-sequencing

As discussed above, in the brain of patients with sepsis, pro-inflammatory microglia predominate. The classical pro-inflammatory genes (e.g., Lcn2, Ccl3, Cxcl13, Ccl5, Il1b, Tnf, and Ccr5) were significantly increased (Fig. 2) [51, 53]. A recent study performed whole-genome RNA-sequencing analysis of brain microglia following an intra-peritoneal (i.p.) LPS challenge at different time points revealed a time-dependent difference in microglial transcriptional profiles [51]. The genes that were significantly upregulated after 6 h of LPS challenge included Itgax, Ch25h, Sppl, Tnf, Saa3, Tnfaip3, and Cd40. Axl, Marco, and Tspo showed delayed elevations, 24 or 48 h after LPS injection. Notably, Itgax, Ch25h, Spp1, and Saa3 were upregulated in the acute phase, which might be related to the activation of microglia, while pro-inflammatory microglia (M1-like) exhibited DAM-like features through the upregulation of Ch25h, Tnf, and Tnfaip3 [72–74]. Axl and Marco showed delayed elevation compared to genes upregulated in the acute-phase after LPS challenge. Axl is known to have the potential to enable macrophage/microglia to rapidly recognize and engulf apoptotic cells [75]. Marco is important for antigen uptake during microglial maturation [76]. The expression of phagocytic genes, such as Trem2, Tyrobp, and Ctsd, was also downregulated after LPS challenge [53]. This appears to indicate the appearance of unique proinflammatory microglia subclass during the acute phase of sepsis, and the phagocytic ability of this cell population is atrophied or diminished. This signature might be microglia-specific as they are not revealed in LPS-activated peripheral immune cells [53]. This CD11b^+^CD45^int^ subset scarcely expressed markers for monocytes (Ly6c and Ccr2) or macrophage (Cd206), suggesting that the appearance of this microglia subset is not due to the contamination from other immune cell types, but due to the intrinsic property of microglia [53].

The SAE-associated microglia cluster significantly decreased the expression of typical microglia signature genes such as Olfml3, Fcrl3, Tmem119, and P2ry12 [54], and downregulated the expression of anti-inflammatory genes such as Mrc1, Arg1, and Ym1 [71], suggesting that a diminished anti-inflammatory capacity of microglia (Fig. 2). Notably, M2-like microglia possess a transcriptional profile marked by Arg1, MR, and Ym1 expression [45]. This suggests that M2-like microglia are non-dominant when compared with M1-like microglia in the brain of SAE. However, it should be clear that by 7 days post-LPS challenge, microglia regained steady-state transcriptomic profiles [54]. The expression of most mRNAs was restored to pre-challenge quiescent levels. The appearance of pro-inflammatory microglia might be a stress response associated with the systemic inflammatory response in sepsis, and this pro-inflammatory activation might phenotypically return to a resting state over time. This suggests microglia have the ability to restore themselves to homeostasis in an inflammatory environment. Nonetheless, whether this event is associated with a parallel expression of M2-like gene remains unclear.

The upregulated genes in microglia after LPS challenge include ApoE, Axl, Cst7, Itgax, and Lgals3 (Fig. 2), which are known to be a part of common neurodegenerative and disease-related signature that has been observed upon several diseases such as AD and PD [54, 77]. Endotoxin-induced neuroinflammation and neurodegeneration are commonly inextricably linked. Transcriptomic analysis of microglia in mouse models of AD and in human AD has revealed a pro-inflammatory signature in Aβ-plaque-associated microglia [78]. Furthermore, gene expression restricted to neurodegeneration was also detected in the upregulated gene Aβ-plaque-associated pro-inflammatory microglia [78]. This appears to suggest a unique microglia response towards neurodegeneration that directs to underlying transcriptional program. The hypothesis of endotoxin contributes to neuronal degeneration remains unproven, however, endotoxin promotes aggregation of amyloid β and tau proteins, suggesting the possibility that endotoxin synergises with different aggregable proteins to cause different neurodegenerative diseases [79]. In addition, in shemer's study, we noticed that neurodegeneration-associated genes were expressed in a time-dependent manner after LPS challenge [54]. ApoE trrand Axl showed delayed increase 24 or 48 h after LPS challenge. This might suggest that more reactive microglia emerge in the progress of disease. For example, in a mouse AD model, two distinct reactive phenotypes were identified at later phase of neurodegeneration, distinct from the early inflammatory phenotype [80].

### Analysis of Driving Forces behind Microglia Phenotypic Change in Neuroinflammation

The core process of neuroinflammation is activation of microglia. This heterogeneous cell population mainly exhibits a pro-inflammatory phenotype in acute phase and a possible neurodegenerative phenotype in later phase in the context of systemic inflammatory response. Through retrieval, three possible reasons are summarized to explain this transition.

Exposure to injury or inflammation is the main factor that induces heterogeneous differentiation of microglia in the context of disease progression. As discussed above, PAMPs and/or DAMPs initiate the primary innate immune response to trigger the subsequent activation of microglia. Inflammation and/or injury are dynamic, exhibiting temporal and spatial heterogeneity [57]. Microglia in an inflammatory environment can express multiple cytokine receptors, which are activated by proinflammatory and restorative cytokines simultaneously, thereby inducing microglia phenotypic transitioning to a transitional phenotype [81]. In the adult brain, microglia express a variety of immune receptors that enable various specific functions. For example, microglia express PRRs (TLRs, NOD-like receptors, C-type lectin receptors, and nucleic acid receptors) that recognize and monitor DAMPs and/or PAMPs (Table 1), enabling rapid activation of microglia and phenotypic transitioning [82–98]. Chemokine receptors (e.g., CX3CR1, CXCR4, CCR1, and CCR2) assist in the migration and positioning of microglia, enhance phagocytic ability, regulate the inflammatory function of microglia, and promote microglial heterogeneous transitioning. Immune receptors (CD33 and TREM2) regulate the degree and duration of microglial activation (Table 1) [99–103]. Injury-induced inflammatory processes are dynamic and exhibit spatial and temporal heterogeneity. By recognizing DMAPs and/or PAMPs, microglia are induced to transition to different functional states.

**Table 1:** Pattern recognition receptors expressed on microglia

PRRs	Member	Reference	Functions in activation of microglia
**Toll-like receptors**	TLR1/2	83	Promote M1-like phenotype; Upregulate NF-κB, TNF-α and IL-1β (by MyD88-dependent manner)
	TLR2	84;85	Regulate microglial activity; Lead to neuronal damage (by MyD88)
	TLR3	86	Upregulate COX-2, mPGES-1 synthesis and prostaglandin E2
	TLR4	87;88;89;90	Regulate microglial activity; Participate in Nurr1 and NF-κB signaling; Promote M1-like phenotype
	TLR9	91;92	Activate microglia; Promote neuronal toxicity; Recognize nucleic acids and signals (by MyD88); Promote M2-like phenotype
**NOD-like receptors**	NLRC3	93	Regulate microglia growth, development and proliferation; Activate microglia (by TLR recognition)
	NLRP3	94;95	Promotes DAMP recognition (by PRR); Activate microglia
	NLRP12	96	Decrease neuroinflammation; Neural protective
**C-type lectin receptors**	Clec7A (Dectin-1)	97;98	Key marker for DAM (only express on postnatal mouse brain); Decrease microglial activity and neuroinflammation
**Nucleic acid receptors**	TLRs,RLRs,DNA sensors	99	Response to cellular damage or inflammation; Act as a cytosolic DNA sensor; Recognize DAMPs and promotes M2-like activation
**Immune receptors**	TREM2	100;101;102;103	Enhance phagocytosis; Decrease neuroinflammation; Mediate and regulate the microglial phenotypic switch; Reduce pro-inflammatory cytokines (e.g., iNOS, TNFα, IL-6, and NO)
	CD33	104	Synergism of CD33 and Trem2 for inhibiting neuroinflammation and activation of microglia

Abbreviations: *COX-2*, cyclooxygenase; *DAM*, disease-associated microglia; *DAMP*, damage-associated molecular pattern; *IL*, interleukin; *iNOS*, inducible nitric oxide synthase; *mPGES-1*, membrane-bound prostaglandin E2synthase1; *MyD88*, myeloiddifferentiationfactor88; *NF-κB*, nuclear factor κB; *NO*, nitric oxide; *PRR*, pattern recognition receptor; *RLRs*, retinoic acid-inducible gene I-like receptors; *TLR*, toll-like receptor; *TNF-α*, tumor necrosis factor α; *TREM2*, triggering receptor expressed on myeloid cells-2

On the other hand, increasing evidence has suggested that high-energy depletion followed by inflammation mediates morphological and functional changes in microglia. Under hypoxic conditions, immune cells exhibit the ability to switch from oxidative phosphorylation to aerobic glycolysis. Increased production of ATP triggers microglial metabolic programming, which boosts both glycolysis and oxidative phosphorylation. Mitochondrial pathway of glycolysis over catabolism is preferentially activated to conserve and generate the metabolic resources necessary to meet cell proliferation and activation needs. Thus, functional modifications of metabolic reprogramming allow microglia to respond with appropriate function in specific situations. Changing the metabolic program from a repair-promoting function to inhibition-dominated function enables its distinct role in different contexts [104, 105]. Studies have shown early metabolic activation of microglia after the systemic inflammatory response via AMP-activated PK (AMPK) and mTOR pathways [106, 107]. Inhibition of ATP-related microglial activation by AMPK attenuates IL-1β and IL-18 production which are commonly observed in macrophages [107]. Furthermore, excessive ATP production increases microglial adenosine production in a negative feedback manner and inhibits neuronal activity through the adenosine receptor A1R during the initial phase of the systemic inflammatory response to sepsis [108]. Thus, a microglia-driven negative feedback mechanism is necessary to maintain neuronal homeostasis.

Finally, neurodegenerative molecules may be involved in the heterogeneous transition of microglia in septic neuroinflammation. Microglia trigger a special sensory mechanism to monitor neural tissue damage in neurodegenerative diseases. However, no reports have recently focused on the connection between neurodegenerative disorders and the acute phase of neuroinflammation after sepsis. Neurodegeneration-associated molecular patterns (NAMPs) are commonly assessed to determine the activation of microglia in AD [109]. The heterogeneous microglial transition is a continuous process, and specific molecules are involved. For example, during the transition from proinflammatory M1-like phenotype to restorative M2-like phenotype, microglia highly express TREM2. However, it was almost undetectable during the transition from the normal stage to the disease-associated stage [110]. Strikingly, unresolved acute neuroinflammation following sepsis may lead to chronic neuroinflammation and cause neurodegenerative diseases. Future research needs to be focused on neurodegenerative damage caused by sepsis to discover the mechanism and extent to which systemic inflammation induced by sepsis affects microglial diversity in the CNS.

## The Main Consequences and Mechanisms Associated with Neuroinflammation after Sepsis

Cerebral dysfunction in SAE reflects systemic metabolic, inflammatory and hemodynamic disturbances following systemic inflammatory response. Despite the lack of specific pathological standard to characterize SAE, a high number of neuroinflammation-associated brain injury outcomes support the occurrence of brain damage and long-term neurological sequelae. These adverse effects may originally result from the dysfunction of neuron and/or synapse, which received persistent stimulation during the extensive activation of immune cells. In addition, adverse events such as hemorrhage or ischemia or BBB disruption caused by cerebrovascular dysfunction also exist in the pathological process of SAE, but extensive neuroinflammation can multiply this risk. In this section, we summarize the main consequences of SAE and the mechanisms associated with those events in the SAE brain.

### Neuronal Dysfunction: Mitochondrial Dysfunction

Neuron is supported by the continuous supplement of glucose and oxygen from circulating blood. Cellular metabolism and neuron function can be altered by the stimulation of activated immune cells and inflammatory mediators in SAE. This alteration could finally impair the function of the brain and influence SAE patient’s long-term morbidity and mortality [111].

**Fig. 3:**
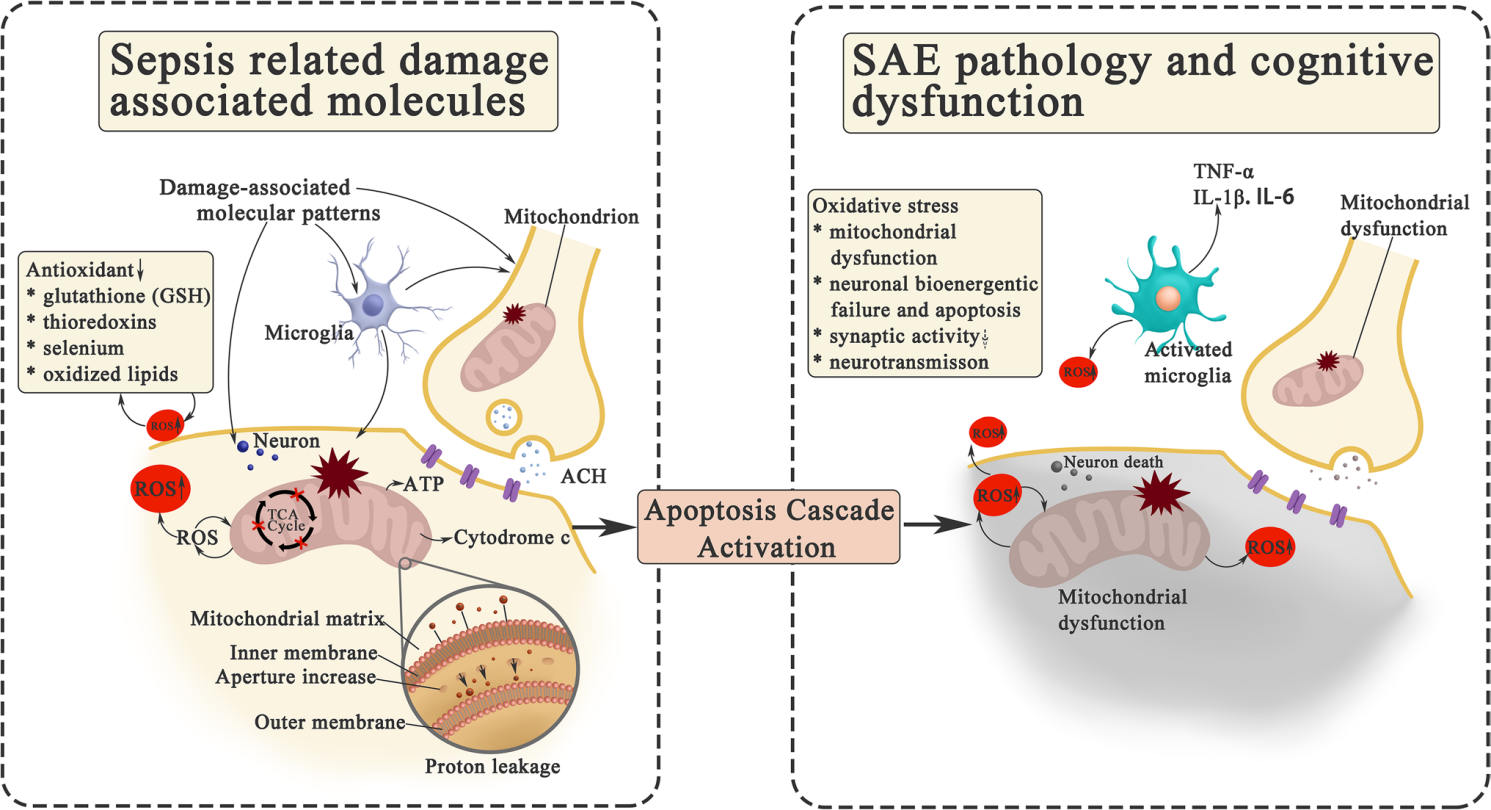
Mechanisms of neuronal mitochondrial dysfunction and brain dysfunction in SAE. Neurons hyperexcited by recognizing DAMPs, followed by the attack of activated microglia and inflammatory cytokines, functional disorder and metabolic disturbances emerge. Due to the extensive and systemic inflammatory response, cells receive insufficient oxygen and are unable to meet their metabolic needs, which leads to  an excessive generation of ROS and RNS. Neuronal mitochondrial dysfunction occurs immediately. The basis of abnormal mitochondrial function in neurons is characterized by increased mitochondrial inner membrane aperture, reduced ATP synthesis, and impaired respiratory chain. The subsequent disturbance of energy supply in neurons and the release of mitochondrial cytochrome c activate neuronal apoptosis cascade. Decreased antioxidant enzymes and increased mitochondrial ROS are lethal to neurons. This could disrupt the process of synaptic transmission (neurotransmission) and synaptic activity. At the same time, DAMPs are recognized by microglia, activating to secrete pro-inflammatory cytokines (e.g., iNOS, TNFα, IL-6) to facilitate neuroinflammation and accelerate brain damage. Abbreviations: ACH, acetylcholine; ATP, adenosine triphosphate; IL, interleukin; RNS, reactive nitrogen species; ROS, reactive oxygen species; TCA, tricarboxylic acid cycle; TNF-α: tumor necrosis factor α

Through reviewing research findings in animals and human beings, one major hypothesis could be proposed to give reasons for these alterations, which is attributed to neuron mitochondrial dysfunction. During sepsis, extensive peripheral immune cell infiltration leads to cerebral vascular dysfunction and hypoperfusion, which contributes to a hypoxic microenvironment in the brain. Insufficient oxygen supplement could not meet the needs of cellular metabolism, leading to increased production of reactive oxygen species (ROS) and reactive nitrogen species (RNS) and actively accelerating inflammation. Uncontrolled generation of mitochondrial ROS and RNS have deleterious effects on cellular functions. In septic animals, the level of intracellular free Ca^2+^ increases, inducing altered membrane structures on neuronal mitochondria [112], impaired ATP synthesis and decreased activity of mitochondrial respiratory chain (Fig. 3) [113]. This might lead to the translocation of mitochondrial matrix, such as cytochrome c, which provokes neuronal apoptotic cascade [114]. Due to this deficit, neuronal metabolic needs cannot be met, followed by overwhelming cell death, leading to acute brain dysfunction after sepsis [114]. Disease-dependent loss of mitochondrial function altered metal homeostasis, and increased ROS production associated with reduced antioxidant defenses directly affect synaptic activity and neurotransmission between neurons, which could eventually lead to the long-term cognitive dysfunction in SAE.

Almost all mitochondrial functions are directly or indirectly linked to the functioning of the oxidative phosphorylation machinery [115]. The reduction in oxidative phosphorylation efficiency is characterized by a sharp decrease in the adenosine 5'-diphosphate/oxygen ratio in the brains of septic animals [112]. Studies have shown that the brain homogenate of septic animals consume more oxygen in the absence of adenosine 5'-diphosphate [112]. Moreover, cytochrome c physiologically binds to endogenous nitric oxide (NO), and competes with oxygen to regulate mitochondrial function [116]. In contrast, the cytochrome levels decrease and cytochrome c oxidase (CcO) activity is reduced during sepsis. CcO is a highly regulated enzyme that is thought to play a critical role in mitochondrial oxidative metabolism and ATP synthesis [115]. CcO is the rate-limiting enzyme in the respiratory chain. Substantial evidence has suggested that CcO dysfunction is frequently associated with increased mitochondrial ROS production and cytotoxicity. Furthermore, LPS induces succinate accumulation, and succinate dehydrogenase rapidly oxidizes this succinate to generate fumarate in a process that requires coenzyme Q (CoQ), which is consumed after LPS stimulation to drive massive ROS production via reverse electron transfer through mitochondrial complex I [117].

### Decreased Learning and Memory Ability: Synaptic Dysfunction

Synaptic dysfunction has been considered as a characteristic feature at late stage neurodegeneration. It has been heralded as a major event in different kinds of neurological diseases with cognitive impairment, such as delirium and dementia [118, 119]. Nonetheless, sepsis is characterized by an extensive systemic immune response to infection. Acute inflammation influences the function of the brain but does not lead to neurodegeneration-like symptoms. Interestingly, cognitive decline is commonly reported on sepsis or septic shock survivors, especially on those who escaped other detrimental effects of sepsis. One main reason for survivor mental decline may be damage caused by neuroinflammation or synaptic plasticity impairment of the hippocampus [120]. The core of synaptic dysfunction is loss of synaptic plasticity and synapses themselves, which is considered to be closely associated with cognitive decline [121]. During sepsis, different kinds of stress-related mediators trigger a vicious cycle of proinflammatory activation of microglia, neuronal apoptosis, and astrocyte-synaptic signalling disruption, leading to synaptic dysfunction. During neuroinflammation, the complement system can be activated to promote the phagocytosis of microglia, leading to inappropriate pruning of synapses [122]. The phagocytosis of microglia on neuronal dendritic spines was confirmed by the occurrence of early inflammation in the brain after LPS challenge [123]. Moreover, global changes in the number of synapses and postsynaptic receptors occur early in the disease process, and these changes correlate most with cognitive decine [121]. Acute neuroinflammation induced by LPS results in excessive microglial activation, which has been found to be consistent with decreased postsynaptic receptor levels [124]. The current study confirms that the signaling of postsynaptic receptor (e.g., GABAergic receptor) may be a key therapeutic target for alleviating abnormal synaptic activity in the medial prefrontal cortex induced by neuroinflammation [124]. On the other hand, evidence connecting proinflammatory events with cognitive decline is supported by hippocampal transcriptome research, which has shown that the expression of immune- and stress-related genes are dramatically increased in the earlier stages post sepsis and continue to be elevated during recovery; however, neurogenesis and myelination as well as related responses are delayed during this period [125]. Cognitive dysfunction induced by acute neuroinflammation is related to molecular abnormalities in the hippocampus. Anti-inflammatory drug and exercise training control of hippocampal signaling factors (e.g., insulin-like growth factor-1 receptor) and neuroinflammatory markers (IL-1β, TNF-α) has been shown to reverse a series of adverse events caused by neuroinflammation, including apoptosis, neural disorganization, and hippocampal oxidative stress [126, 127]. Given the importance of recognizing exercise training as complementary therapy in controlling the acute neuroinflammation in the hippocampus, future studies are warranted.

Currently, increasing evidence supports the idea that glial cells influence the normal state and function of synapses in the healthy brain. Therefore, the pathological transformation of glial cells may contribute to synaptic and cognitive impairment in the brain [128]. The cytokine production during sepsis magnifies glutamate-induced glial cell dysfunction, producing detrimental effects on astrocyte reactivity and synaptic plasticity. These outcomes have been demonstrated to precede the development of irreversible neurodegenerative pathology. Therefore, given the synaptic plasticity in the healthy brain, mechanisms involved in synaptic dysfunction that are associated with cognitive impairment need to be further studied to provide strategies for clinical treatment in the future.

### Cerebrovascular Damage: Ischemia, Hemorrhage, and BBB Disruption

In post-mortem studies, septic shock was associated with a high prevalence of ischemic neurons and hemorrhage, especially in areas that are susceptible to hypotension and hypoxia [30]. A decreased cerebral blood flow (CBF) was demonstrated in septic shock [129]. One possible reason is impaired cerebrovascular autoregulation. Another is extensive and persistent inflammation of cerebrovascular ECs.

#### Loss of Functional Cerebrovascular Autoregulation

Cerebral autoregulation plays a pivotal role in modulating CBF. The ability of cerebrovascular autoregulation leads to an average arterial pressure of CBF in a steady range 60 to 150 mmHg [130]. Once this function is dysregulated, sepsis can exacerbate disrupted cerebral perfusion via vasodilation and changes in microcirculation, which might be associated with cognitive impairment in sepsis survivors [10]. Approximately 11% of sepsis or septic shock patients develop ischemic stroke [131]. The risk of sepsis-associated ischemic stroke is increased, particularly in patients with atrial fibrillation [132]. A study based on single-photon emission computerized tomography (SPECT) scanning and magnetic resonance imaging (MRI) confirmed frontal and parietal cerebral perfusion abnormalities in patients with delirium [133, 134]. Patients with ischemic stroke have been reported to present with long-term neurological sequelae, which are thought to impair functional activation of cerebral vascular ECs [135]. In fact, one-half of patients with sepsis present with brain dysfunction associated with the destruction of cerebral autoregulation [136]. One hypothesis has suggested that the disruption to cerebral autoregulation may contribute to brain hypoperfusion and neuronal damage. Cerebral hypoxia is undoubtedly one of the primary mechanisms leading to brain dysfunction after sepsis [136]. Hypoxia-related damage may be caused by overexpression of peripheral inflammatory mediators, resulting in dysfunctional activation of cerebral vascular ECs. These abnormalities may be the main reasons for the formation of thrombotic occlusion in capillaries and impaired cerebral blood perfusion and microcirculation [137, 138]. In addition, brain hemorrhage has been reported in patients who have died from septic shock [62, 139]. Subtle brain hemorrhage might contribute to microbleed syndrome, associated with progressive decline in cognitive function [140].

#### Blood–brain Barrier Disruption

The BBB separates the CNS from blood circulation, preventing foreign substances from entering the brain. Functionally, the BBB regulates the microenvironment and maintains CNS homeostasis. During sepsis, excessive or persistent systemic inflammation disrupts the BBB, enabling the brain to be flooded with proinflammatory mediators [141]. A large number of cytokines enter the brain to activate microglia and other glial cells to trigger neuroinflammation.

A popular hypothesis of BBB disruption after sepsis involves EC dysfunction. Under this theory, cytokines activate microglia in the brain after sepsis; however, the activation of ECs occurs almost simultaneously or even earlier [142, 143]. The proinflammatory microglia facilitate ECs’ acquisition of proinflammatory phenotype, stimulating continuous release of cytotoxic cytokines, ultimately leading to EC apoptosis [144]. As discussed previously, microglia proliferation and EC crosstalk reduce new blood vessel formation and promotes tissue repair. However, excessive activation exacerbates EC death and dysfunction. EC dysfunction is considered to be a major cause of BBB disruption after sepsis. In addition, prothrombotic ECs can activate the anticoagulation cascade, leading to impaired BBB integrity, tissue hypoperfusion, and ischemic stroke [145, 146]. Excessive EC apoptosis and enhanced EC-associated coagulation cascade activity powerfully promote increased BBB permeability and trigger a series of acute cerebral dysfunctional changes, causing neuronal damage and brain edema [147, 148]. The production of ROS and RNS perpetuates the vicious cycle of increased BBB permeability [148, 149]. During sepsis, cytokine storm-mediated microglia activation and endothelial phenotypic changes stimulate the production of ROS and RNS, thereby increasing the permeability of the BBB and impairing its macromolecules (e.g., occludin and zonula occludens 1) [148, 150, 151]. ROS and RNS regulate innate immunity through direct damage or activation of the transcription factor NF-κB, enhancing the production of TNF-α, IL-1β, IL-6, IL-8, IL-17, and IL-18 to modulate vascular EC dysfunction during sepsis [152].

Moreover, the systemic inflammatory response to sepsis causes more peripheral immune cells to translocate and adhere to the BBB, accelerating BBB destruction. During the early phase of neuroinflammation, the role of sepsis-related endothelial activation is considered beneficial because it inhibits cytotoxic cytokine diffusion and orchestrates leukocyte recruitment. However, intensive inflammatory stimulation induces ECs to differentiate into pro-adhesive ECs [153]. This EC phenotype significantly regulates the process of leukocyte adhesion. A study confirmed cerebrovascular leukocyte–EC adhesion after sepsis, and this adhesion plays an important role in BBB disruption [154]. This type of EC increases the expression of ICAMs, including ICAM-1, ICAM-2, VCAM-1, and PECAM [152]. The leukocyte ligands then adhere to ECs and direct migration toward chemotactic mediators cause broader endothelial inflammation. Inhibiting the adhesion of leukocyte–ECs can significantly mitigate BBB permeability [154].

From another perspective, activation of the complement system plays a significant role in sepsis-related BBB leakage. C5a anaphylatoxin has been characterized as a critical mediator of sepsis and septic organ dysfunction [155, 156]. Following sepsis, many cytokines infiltrate the brain, with the expression of cerebral endothelial C5a receptor upregulated in a time-dependent manner [157]. Following C5a expression upregulation, microglia are recruited and induced to release proinflammatory cytokines, and their phagocytic capacity is enhanced; astrocytes are also activated [158]. Moreover, sepsis is associated with persistent astrocyte and microglia activation. The upregulation of C5a expression activates the stress system of the brain. Upregulation of C5a anaphylatoxin expression in the presence of neuronal C5a receptor under inflammatory conditions render the brain more vulnerable to C5a-mediated secondary disruption of the BBB [158]. In fact, blocking C5a expression reduced sepsis-induced neuronal activation in the paraventricular nucleus and amygdala and improved BBB and pituitary function [158].

Finally, the production of matrix metalloproteinases (MMPs) is a recognized mechanism of BBB disruption. In animal models of sepsis, MMP-8 cleaves collagen in the extracellular matrix of the choroid plexus, increasing barrier permeability [159]. Various factors have been shown to promote the expression of MMPs, such as vascular growth factor, IFN-γ, IL-1β, and IL-6 [160]. In addition, the adhesion of immune cells and expression of PAMPs and DAMPs are also reasons that increase MMP expression [152]. Taken together, these actions lead to BBB leakage under inflammatory conditions, which is physiologically harmful to the brain and facilitates cytokine entry into the CNS [150]. However, it remains very challenging to control microglial activation and EC dysfunction, especially since the EC response may impair the BBB to a significant extent.

## Prospective Interventions Targeting Microglia and Neuroinflammation after Sepsis

Specific strategies for clinical management for SAE supported by evidence-based clinical practice are lacking. Clinical management approaches to different kinds of encephalopathy often originate from its causes and cognitive presentation [134, 161]. Early identification and management of potential sepsis gives patients the best chance to prevent cognitive sequelae [162]. The 2021 International Guidelines for the Management of Sepsis and Septic Shock advocated early fluid resuscitation, early source control, and timely use of appropriate antibiotics; however, no SAE management methods were mentioned [163]. Although some compelling evidence-based data on neuroinflammation in SAE have been reported in recent decades, no evidence-based pharmacological treatment is available to support specific neuroinflammation treatment in SAE [164]. This section reviews some potential treatments for neuroinflammation, focusing on microglia and inflammatory processes. Using these mechanisms may improve SAE treatment.

### Potential Therapeutic Approaches Targeting Microglia

Due to the important role of microglia in maintaining brain homeostasis, the potential therapeutic strategies for targeting microglia are promising. As discussed above, microglia play an essential role in the development of neuroinflammation. Inhibiting the activation of microglia by pharmacological or non-pharmacological methods, or indirectly reducing the activation of microglia by preventing infiltration of peripheral cells might be effective therapeutic methods for inhibiting neuroinflammation.

#### Inhibition of Microglial Activity

Recently, the detrimental effects of microglia on disease progression have attracted the interest of researchers, who have been exploring pharmacological or genetic means of attenuating these effects. The development of CNS-penetrating drugs that inhibit colony-stimulating factor 1 receptor (CSF-1R) activity, on which microglia depend for survival, has enabled the study of microglia in both the healthy and diseased CNS [165]. The common inhibitor PLX5622 causes the death of IBA1^+^TMEM119^+^ microglia but has little effect on circulating monocytes. Beneficial effects of neuroinflammation in a mouse model of neurotropic infection were observed when microglia were eliminated by CSF-1R functional inhibition [166]. CSF-1R is a microglia surface receptor with signaling activity in the brain [165]. CSF-1R inhibitor treatment reduced LPS-stimulated neuroinflammation in mouse brains [167]. Furthermore, CSF-1R inhibitors improved cognitive functions, restored lost dendritic spines, and reduced plaque formation in AD models [168, 169]. All these findings provide promising research prospects for microglial inhibitor treatment of neuroinflammation in the brain after sepsis.

#### Intervention with Microglial Molecular Targets

Since microglia activation is a core step in promoting the development of neuroinflammation, it is believed that one of the goals of microglia-targeted therapy is to maintain the homeostatic function of microglia in the inflammatory environment, and to inhibit inflammation or inhibit the promotion of pro-inflammatory microglia (M1-like). Thus, antagonizing the molecular targets of pro-inflammatory activation and agonizing the molecular targets that increase expression of homeostatic and anti-inflammatory signatures are considered feasible molecular therapeutic approaches. On the basis of our understanding of homeostatic and pro-inflammatory microglia (M1-like) signatures, a number of specific microglia molecular targets are beginning to emerge. Targets for antagonizing pro-inflammatory transition include Lcn2, Ccl3, Cxcl10, Ccl5, Il1b, Ccl2, Itgax, Ch25h, Tnf, Tnfaip3, and Cd40 [54]. Targets for boosting function include miR-124, TGFβ, Mertk, Cx3cr1, P2ry12, Mef2c, Sall1, and Mafb [78]. Targets for promoting anti-inflammatory effects and repairing function include Arg1, MR, Ym1/2, Emilin2, Fn1, Alcam, and Cspg4 [23, 45]. Some molecules, such as TGF-β, Axl, and TREM2, play different roles at different stages after LPS challenge, which depends on the progression phase of the disease. Therefore, taking full advantage of these signatures could generate different therapeutic effects at different stages of disease progression.

#### Inhibition of Peripheral Infiltrating Cell

Infiltration of peripheral cells, such as monocytes, plays an important role in early microglial recruitment and late microglial phenotypic shift in sepsis survival. Blocking monocyte recruitment during the acute inflammatory phase after sepsis in CCR2^−/−^ mice reduced other inflammatory responses, including neutrophil recruitment and microglial activation [170]. This study suggested that both neutrophils and CCR2^+^ inflammatory monocytes were recruited at significant levels to the brain during the acute inflammatory phase of sepsis. Treatment with anti-CCR2 monoclonal antibodies (mAbs) blocked the entry of CCR2^+^ monocytes into the brain and thus protected the brain from cognitive impairment [170]. The number of microglia in the late-stage sepsis-surviving brain is increased, and they may shift to a repair phenotype, thereby remaining activated [171]. A study showed that the increased number of these microglia was largely due to self-proliferation. However, this proliferative state was completely abolished in CCR2-knockout mice [171]. Therefore, brain-infiltrating peripheral monocytes may contribute to the functional and phenotypic transitioning of microglia during sepsis.

#### Inhibition of Specific Proinflammatory Pathway

Blocking the deleterious role of microglia in SAE can be more effectively achieved by inhibiting specific proinflammatory pathway activation. The protective effect of IL-1R inhibition has been shown to be beneficial in limiting microglial activation. Disabling IL-1R is sufficient to reduce the proliferation of microglia in the hippocampus and improve cognitive function in LPS-challenged mice [172]. The important role of IL-10 in inducing the reparative transformation of microglia has been previously described. Microglia constitutively express high levels of IL-10R [54], and IL-10R deficiency is critical for preventing microglial hyperactivation during septic shock or bacteremia [54]. Studies have also shown that autophagic flux and the expression of autophagy-related genes in microglia are significantly inhibited by TLR4 activation after LPS exposure, but inhibition of PI3K activity can restore autophagic flux and increase microglial phagocytic activity [173]. Among all the factors that induce microglial activation, regulating inflammation-induced apoptosis, ROS and other free radical production, and the expression of many cytokines can reduce microglial activation and significantly improve cognitive function. In LPS-exposed mice, the lack of NADPH oxidase (NOX) reduces the activation of microglia and confers a protective effect against synaptic dysregulation and cognitive impairment following systemic inflammation [174]. In addition, it has been confirmed that cytosolic receptors of inflammasome proteins are involved in perpetuating neuroinflammation [175]. The activity of microglia attacked by LPS decreases after NLRP3 inhibition, thereby improving cognitive function in mice [176]. NLRP3 may represent a new therapeutic target, the activity of which can be inhibited to protect the brain from toxic peripheral inflammation during systemic infections.

### Therapeutic Strategies for Targeting Peripheral Cytokines into the Brain

As discussed above, cytokines can enter the brain mainly through three pathways to induce the activation of inflammatory cells in the brain and the occurrence of neuroinflammation. Blocking inflammatory signals or cytokine movement before they enter the brain is considered a feasible strategy for treating neuroinflammation. First, this blockade strategy can protect against BBB leakage after the onset of sepsis. Most recent research has focused on BBB disruption, and more interventions are being tested to modulate BBB permeability. Treatment with MMP-2 and MMP-9 inhibitors or MMP-2 or MMP-9-specific inhibitors in a rat model of sepsis reversed MMP-induced cognitive changes to BBB permeability, brain inflammatory responses, and sepsis [177]. In addition, MMP8 and MMP9 inhibitors have been shown to have significant effects on improving BBB permeability in mice after sepsis [178]. Occludin is a crucial structural component of the BBB. Regulation of occludin protein functions was also found being sufficient to modulate BBB permeability [179].

Exercise training has been shown to have the ability to control the inflammatory cytokine storm that enter the brain. Aerobic exercise and resistance exercise training can reverse adverse events caused by neuroinflammation, protecting neurons, avoiding hippocampal tissue from karyopyknosis, and reducing cognitive dysfunction [127, 180]. Also, recent systematic review and meta-analyses clearly indicate that exercise training is a powerful strategy for boosting the systemic immune response to fight against inflammatory and viral diseases [181].

Recent studies have demonstrated the efficacy of ultrasound-guided transcutaneous stimulation of the vagus nerve in reducing systemic inflammation, modulating hippocampal microglial activity, and restoring LPS-induced memory deficits in mice. The vagus nerve is a typical immunomodulatory reflex circuit with afferent projections that sense inflammatory changes and that can reduce proinflammatory cytokine production [182]. The vagus nerve has been reported to induce acetylcholine release through a subset of ChAT-expressing T lymphocytes, thereby inhibiting TNF-α production and downregulating proinflammatory gene expression [183]. Inhibition of this innate immune response requires the α7 subtype of the nicotinic acetylcholine receptor (α7nAChR), which can be activated by pharmacological agonists or by direct electrical stimulation [184]. Plasma levels of TNF-α can be reduced by stimulating the vagus nerve [182]. These findings suggest that vagal nerve stimulation can exert an anti-inflammatory effect. In addition, brain-resident microglia and circulating macrophages express α7nAChR [185]. In infection and stroke-induced neuroinflammation, cholinergic agonists affect microglial activation, improving cognitive outcomes after trauma and common perioperative complications [186–188]. In general, vagus nerve stimulation is a promising therapeutic measure that provides better and specific therapeutic outcomes and fewer side effects than currently used clinical treatments.

### Anti-inflammatory Drugs and Inflammatory Inhibitors Targeting Neuroinflammation

Corticosteroids, especially dexamethasone, are used in research and treatment of SAE due to their anti-inflammatory and immunosuppressive properties. Studies have shown that a small dose of dexamethasone can regulate neuronal autophagy after sepsis and protect rat cerebral cortical neurons [189]. In vitro studies showed that dexamethasone inhibits the secretion of pro-inflammatory factors and chemokines in microglia activated by LPS, and reduces the neuro-inflammatory response and migration of BV-2-like microglia playing a neuroprotective role against inflammation [190].

Other well-studied inflammatory inhibitors include minocycline [191], vitamin C [192], TGFβ1 [193], rapamycin [194], and other synthetic drugs [195]. However, although some anti-inflammatory drugs have been shown to reduce neuroinflammation, few have direct functional effects on microglial activity. In addition, even though the benefits of inflammatory inhibitors on neuroinflammation in SAE has been proven, clinical trials failed to observe significant benefits in cognitive impairment yet [196], so more effective treatments are urgently needed.

## Conclusion

SAE is a severe complication of sepsis. Brain-resident immune cells followed by peripherally immune cells mutually constitute the brain’s immune network. The cytokine storm initiated by systemic inflammatory response can enter the brain to aggravate neuroinflammation, which mainly depends on hormone pathways, cellular pathways, and neuro-endocrine pathways. As a homeostasis maintainer in the brain, microglia were initially shifted to the population dominated by pro-inflammatory microglia (M1-like) in the context of systemic inflammatory response during sepsis. The essence of this heterogeneous process is the differentiation of gene expression, generally manifested as upregulation of pro-inflammatory genes and down-regulation of homeostatic genes and anti-inflammatory genes during neuroinflammation. Moreover, upregulation of neurodegenerative genes is also found in this process, suggesting that SAE may induce secondary neurodegeneration. The spatial continuity of neuroinflammation can be the reason for adverse events induced by brain injury and encephalopathy, including neuronal dysfunction, decreased learning and memory abilities, and impaired cerebrovascular function. Cognitive decline and mental abnormalities are the main manifestations of SAE patients; however, they are prone to be neglected. Uncontrolled neuroinflammation may cause more severe sequelae in survivors. Therefore, developing available drugs or therapeutic strategies to inhibit microglia activation and intervening or blocking neuroinflammation can suppress further adverse events caused by neuroinflammation and provide effective therapeutic strategies for improving the prognosis for SAE patients.

## Data Availability

Not applicable.

## Abbreviations

AD: Alzheimer's disease
ATP: Adenosine triphosphate
AMPK: AMP-activated PK
Arg1: Arginase1
BBB: Blood–brain barrier
C5a: Complement component 5a
CBF: Cerebral blood flow
CCL: C–C motif chemokine ligand
CcO: Cytochrome c oxidase
CD: Cluster of differentiation
CLP: Cecal ligation and perforation
CNS: Central nervous system
CoQ: Coenzyme Q
CSF-1R: Colony-stimulating factor 1 receptor
CXCL: Chemokine (C-X-C motif) ligand
CXCR: Chemokine (C-X-C motif) receptor
DAM: Disease-associated microglia
DAMP: Damage-associated molecular pattern
EC: Endothelial cell
GABA: Gamma-aminobutyric acid
HMGB1: High mobility group box 1
HSPA5: Heat shock protein family A (Hsp70) member 5
ICAM: Intracellular adhesion molecule
IFN: Interferon
INOS: Inducible nitric oxide synthase
IL: Interleukin
LPS: Lipopolysaccharide
LTP: Long-term potentiation
MMP: Matrix metalloproteinases
MR: Mannose receptor
MRI: Magnetic resonance imaging
MTTFA: Mitochondrial transcription factor A
NADPH: Nicotinamide adenine dinucleotide phosphate
NAMP: Neurodegeneration-associated molecular patterns
NF-κB: Nuclear factor κB
NLRP3: Recombinant NLR family, pyrin domain containing protein 3
NO: Nitric oxide
NOD: Nucleotide-binding oligomerization domain
NOX: NADPH oxidase
NK cell: Natural kill cell
PAMP: Pathogen-associated molecular pattern
PECAM: Platelet endothelial cell adhesion molecule
PI3K: Phosphatidylinositol-3-kinase
PRR: Pattern recognition receptor
RLR: Retinoic acid-inducible gene I-like receptor
RNS: Reactive nitrogen species
ROS: Reactive oxygen species
SAE: Sepsis-associated encephalopathy
SIRS: Systemic inflammatory response syndrome
SPECT: Single-photon emission computerized tomography
SQSTM1: Sequestosome 1
SR: Scavenger receptor
TBI: Traumatic brain injury
TGF: Transforming growth factor
TLP: Toll-like receptor
TNF-α: Tumor necrosis factor α
TREM2: Triggering receptor expressed on myeloid cells-2
VCAM-1: Vascular cell adhesion protein-1
Ym1: Chitinase-3-like-3
α7nAChR: α7 Subtype of the nicotinic acetylcholine receptor
